# Collective Intelligence–Based Participatory COVID-19 Surveillance in Accra, Ghana: Pilot Mixed Methods Study

**DOI:** 10.2196/50125

**Published:** 2024-08-12

**Authors:** Gifty Marley, Phyllis Dako-Gyeke, Prajwol Nepal, Rohini Rajgopal, Evelyn Koko, Elizabeth Chen, Kwabena Nuamah, Kingsley Osei, Hubertus Hofkirchner, Michael Marks, Joseph D Tucker, Rosalind Eggo, William Ampofo, Sean Sylvia

**Affiliations:** 1 Department of Health Policy and Management, University of North Carolina Chapel Hill, NC United States; 2 Department of Social and Behavioral Sciences, School of Public Health, University of Ghana Accra Ghana; 3 Department of Health Behavior, Gillings School of Global Public Health, University of North Carolina Chapel Hill, NC United States; 4 Cognate Systems Company Limited Accra Ghana; 5 Prediki Prediction Markets GmbH Vienna Austria; 6 Clinical Research Department, London School of Hygiene and Tropical Medicine London United Kingdom; 7 Division of Infection and Immunity, University College London London United Kingdom; 8 Institute for Global Health and Infectious Diseases, University of North Carolina Chapel Hill, NC United States; 9 Noguchi Memorial Institute of Medical Research, University of Ghana Accra Ghana

**Keywords:** information markets, participatory disease surveillance, collective intelligence, community engagement, the wisdom of the crowds, Ghana, mobile phone

## Abstract

**Background:**

Infectious disease surveillance is difficult in many low- and middle-income countries. Information market (IM)–based participatory surveillance is a crowdsourcing method that encourages individuals to actively report health symptoms and observed trends by trading web-based virtual “stocks” with payoffs tied to a future event.

**Objective:**

This study aims to assess the feasibility and acceptability of a tailored IM surveillance system to monitor population-level COVID-19 outcomes in Accra, Ghana.

**Methods:**

We designed and evaluated a prediction markets IM system from October to December 2021 using a mixed methods study approach. Health care workers and community volunteers aged ≥18 years living in Accra participated in the pilot trading. Participants received 10,000 virtual credits to trade on 12 questions on COVID-19–related outcomes. Payoffs were tied to the cost estimation of new and cumulative cases in the region (Greater Accra) and nationwide (Ghana) at specified future time points. Questions included the number of new COVID-19 cases, the number of people likely to get the COVID-19 vaccination, and the total number of COVID-19 cases in Ghana by the end of the year. Phone credits were awarded based on the tally of virtual credits left and the participant’s percentile ranking. Data collected included age, occupation, and trading frequency. In-depth interviews explored the reasons and factors associated with participants’ user journey experience, barriers to system use, and willingness to use IM systems in the future. Trading frequency was assessed using trend analysis, and ordinary least squares regression analysis was conducted to determine the factors associated with trading at least once.

**Results:**

Of the 105 eligible participants invited, 21 (84%) traded at least once on the platform. Questions estimating the national-level number of COVID-19 cases received 13 to 19 trades, and obtaining COVID-19–related information mainly from television and radio was associated with less likelihood of trading (marginal effect: −0.184). Individuals aged <30 years traded 7.5 times more and earned GH ¢134.1 (US $11.7) more in rewards than those aged >30 years (marginal effect: 0.0135). Implementing the IM surveillance was feasible; all 21 participants who traded found using IM for COVID-19 surveillance acceptable. Active trading by friends with communal discussion and a strong onboarding process facilitated participation. The lack of bidirectional communication on social media and technical difficulties were key barriers.

**Conclusions:**

Using an IM system for disease surveillance is feasible and acceptable in Ghana. This approach shows promise as a cost-effective source of information on disease trends in low- and middle-income countries where surveillance is underdeveloped, but further studies are needed to optimize its use.

## Introduction

### Background

Emerging infectious diseases pose substantial and persistent risks to human and animal health globally. Yet, our ability to monitor these threats is limited, especially in low- and middle-income countries (LMICs) where many infectious disease outbreaks initially emerge [1,2]. Traditional, test-based infectious disease surveillance is expensive and prone to selection bias and involves significant time lags in contexts with weak public health infrastructure. Test-based systems relying solely on recorded case information from individuals seeking medical care tend to underestimate the true disease burden [3]. Underestimation is likely to be exacerbated in settings common in LMICs, where access to formal care is limited. More affordable and reliable methods are required to deliver timely data on disease patterns and inform the response to outbreaks in LMICs as either a supplement or stopgap for test-based methods.

Several low-cost supplementary surveillance approaches have been deployed in recent years [2,4-7]. Key among these are participatory syndromic surveillance systems, such as Influenzanet and Flu Near You. These systems rely on volunteers to regularly report symptoms using brief web-based surveys, mobile apps, interactive voice response, and SMS text messages [8-13]. Participatory disease surveillance systems can, in theory, collect information from a more representative population (including those who do not seek formal care) and can provide near–real-time data on disease trends to enable rapid health response [14,15]. Challenges in outbreak control because of delays in timely data capturing during the COVID-19 pandemic demonstrated the need for participatory disease surveillance systems that can be used in LMIC settings [16,17]. Key challenges documented include low and selective participation, which introduces bias because of a lack of population representativeness [18]. Therefore, data from information market (IM)–based participatory surveillance could be used to complement traditional participatory disease surveillance systems, such as Influenzanet, and reduce the consequential impact of the lack of population representativeness [11].

Participatory disease surveillance systems that incorporate features of IMs could be an approach to circumventing some of these challenges simultaneously. IMs are a crowdsourcing approach that relies on fundamental insights from economics to encourage accurate reporting by participants and efficiently aggregate community beliefs into a single interpretable “now-cast” or forecast. In the simplest design, participants trade (buy and sell) shares in a given outcome (eg, whether or not an event will occur by a specified date), typically in a virtual marketplace. The supply and demand for shares at a given time yield a market-clearing price, which is interpretable as an aggregate index of participants’ perceptions of the likelihood of the event [19]. With active participation, this index fluctuates over time, similar to a stock price, increasing and decreasing as community perceptions evolve. Prediction markets, one form of IMs, have been widely used in industry and government to forecast product sales and make predictions regarding geopolitical events as well as in various other contexts where reliable data are scarce or difficult to collect [20-22].

IM surveillance uses this mechanism to elicit real-time information on community participants’ perceptions of disease trends. In contrast to prediction markets, the objective is not to forecast disease trends into the future but to efficiently elicit reliable perceptions from the community in real time. When designed effectively and with sufficient engagement, IM surveillance systems may prove more reliable than syndromic participatory disease surveillance systems by (1) improving scalability by motivating wide participation, (2) eliminating the need for participants to be representative of a population, (3) automatically encouraging more reporting from key demographic groups or geographic areas, and (4) efficiently aggregating participant information into indices useful for decision makers without additional modeling or confounder adjustment.

IM disease surveillance has not been evaluated in an LMIC context. Two previous studies describe case studies in higher-income countries (the United States and Taiwan) [23,24]. These studies suggest that IM surveillance is feasible and accurate for influenza and dengue fever in these settings [19,23-25].

### Objectives

The objective of this study was to evaluate the approach’s feasibility for a lower-income context. We deployed an IM-based surveillance platform to monitor disease trends and associated socioeconomic indicators in Accra, Ghana, during the COVID-19 pandemic.

## Methods

### Study Design

This pilot study assessed the use of a tailored IM system for disease surveillance in Ghana. In IMs, participants report by trading contracts specifying payoffs tied to a future event [26]. Each participant can buy or sell shares in these contracts based on their expectations. For example, consider a contract that pays US $100 if candidate X wins an election. If the market price of an X contract is currently US $53 and the price of a Y contract (for candidate Y) is currently US $21, we would interpret this to mean that the market “believes” candidate X has a 53% chance of winning and candidate Y has a 21% chance of winning [5]. This approach functions as an information collection and aggregation mechanism: each participant knows and is incentivized to use it to make trades. The market for each contract yields prices that reflect the trader’s expectations of future events [27].

### Study Setting

This pilot study was conducted among residents in Accra City, Greater Accra Region, Ghana. Accra is Ghana’s capital and the largest city and was one of the main COVID-19 epicenters in 2021 [28]. As Ghana’s commercial and political capital, Accra receives visitors from other regions of Ghana and neighboring countries [29]. There are 16.99 million internet users in Ghana as of February 2022, of which 99.3% use smartphones [30].

### Sample Size

The study included individuals aged ≥18 years who had access to a personal desktop or computer and self-reported as frequent internet users (>1 time/wk). Previous studies that used IMs to forecast infectious diseases recruited 40 to 130 participants but observed high attrition rates. Considering the potential for a low participation rate, the study assumed that 105 participants would be sufficient to achieve the pilot study goals of assessing the feasibility and acceptability of the approach. Thus, total enrollment was completed for 50 health workers and 55 community members in Accra.

### Recruitment

An eligibility survey was designed based on gender; age; occupation; device ownership; and digital experience, which this study defined as proficiency in using modern technological devices, especially computers. Two types of respondents were targeted for this study: health workers and volunteer community members. A total of 5 recruiters were trained on the recruitment questionnaire, and participants were purposively recruited until a point of saturation was reached on major issues in the criteria.

Health workers, including medical doctors, nurses, pharmacists, community health workers, local health officials, and laboratory technicians, were purposively recruited from clinics and hospitals in Accra. Community members were purposively recruited in different localities across Accra. Recruiters approached potential respondents in their homes and workplaces. This target included students, teachers, drivers, IT experts, web developers, and computer system engineers. Eligible health workers and community volunteers willing to participate were encouraged to promote the study among their colleagues, friends, peers, and family members. They could refer interested individuals for eligibility screening.

### Data Collection

#### Overview

A total of 105 recruits, including 50 health workers and 55 community members, were invited to participate. The study was pretested among 4 community members and 2 health workers over 3 weeks; learnings were used to improve the questions and some platform features. The main pilot was conducted over 3 months, from October 1 to December 31, 2021. The Prediki platform was used to coordinate trading and interactions with the participants regarding expectations of future COVID-19 cases, deaths, and COVID-19 vaccine uptake in the Greater Accra region and nationwide. The buying and selling of contracts was tied to the future realizations of the outcomes based on regional statistics, as reported by the Ghana Health Service and an independent surveillance system (FluNet). Prices in these markets were used to track expectations of trends over time.

#### Participant Engagement

Between September 15, 2021, and October 1, 2021, all eligible participants (health care workers and community members) willing to participate in the study were recruited until the desired sample size was attained. Recruits completed a web-based baseline survey collecting sociodemographic characteristics, including age, occupation, and a major news source (such as television, radio, and social media). Participants were enrolled in a research group on WhatsApp Messenger (WhatsApp Inc) administered by the study team, where instructional videos and project-related announcements were shared to increase engagement.

### Prediction Market Implementation

**Figure 1:**
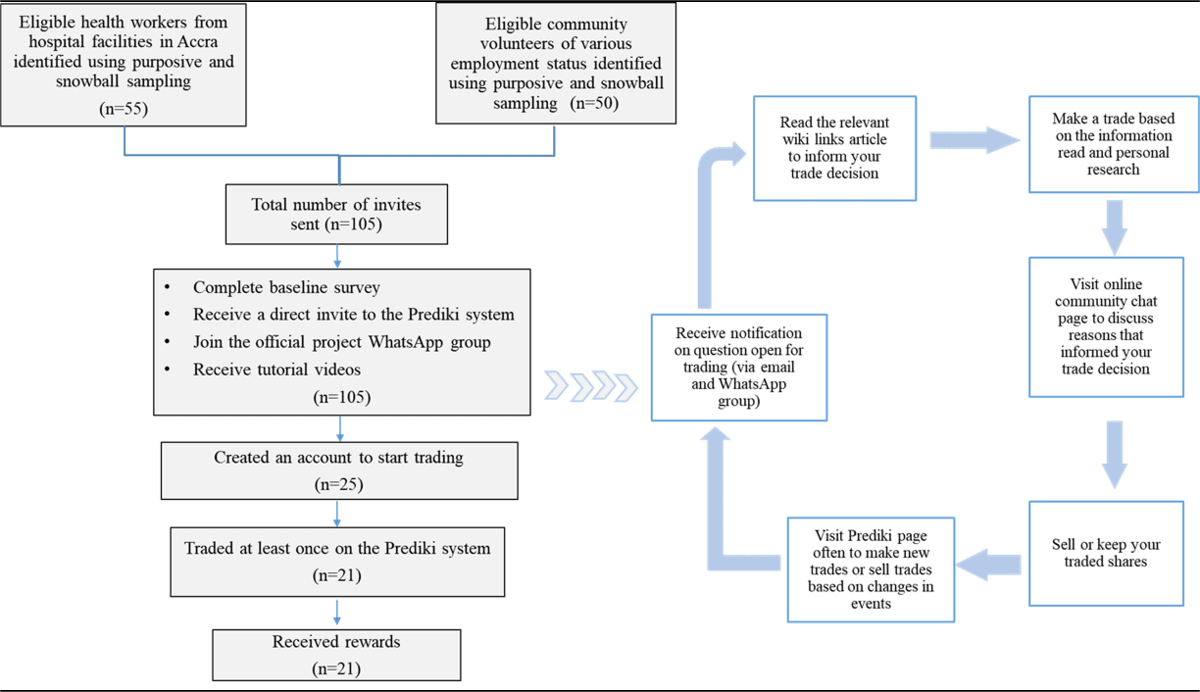
Flowchart showing the step-by-step recruitment process and participation cycle in the COVID-19 prediction markets survey in Ghana in 2021.

Figure 1 shows the step-by-step recruitment process and the stages of participating in the prediction markets survey.

#### Preparation

**Figure 2:**
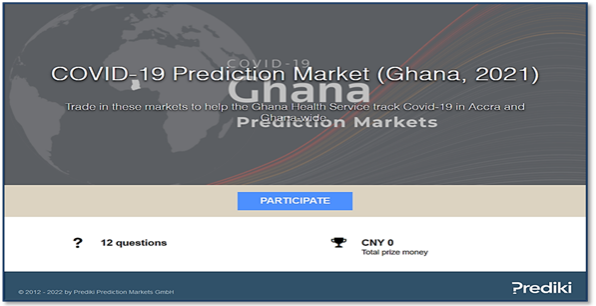
The Prediki prediction markets interface showing the user landing page for the COVID-19 Ghana prediction markets survey.

**Figure 3:**
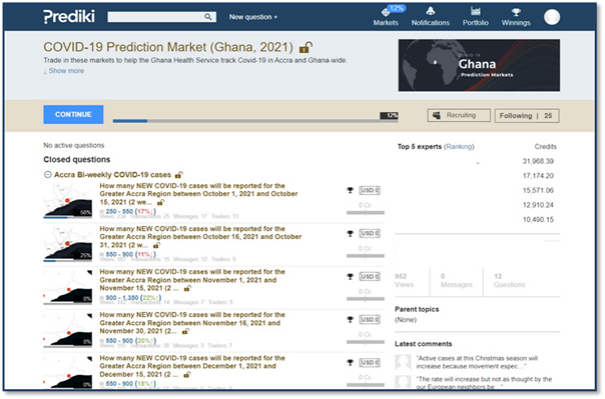
How questions opened for trading are depicted on the user interface of the Prediki markets system. Participants can click on each question to be directed to the trading options.

We used the Prediki Prediction Markets 2.0 system (Prediki Prediction Markets GmbH) to design a COVID-19 prediction market for Ghana (Figure 2). Participants received individual invite links to the Prediki platform and 3 instructional videos on using prediction markets before the prediction markets opened for trading (Figure 3). One video informed participants of the need to help the Ghana Health Service monitor outbreaks and the potential of using prediction markets in crowdsourcing forecasts on COVID-19 (Multimedia Appendix 1). The other videos guided participants on how to make trades on the Prediki platform (Multimedia Appendix 2) and explained the reward mechanism to be used in the study (Multimedia Appendix 3).

#### Trading

**Figure 4:**
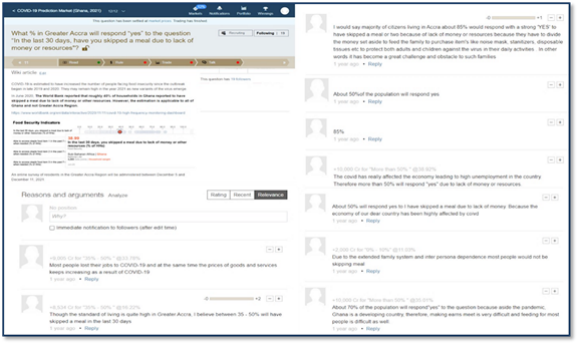
A picture example showing some website links to relevant wiki articles and the community discussions that helped inform participants’ trading decisions and choices based on ongoing events.

The trading on the platform began on October 1, 2021, with 2 sets of question types (Figure 3). Five questions had an end date of December 31, 2021, and were open for trading from October 1 to December 31, 2021. The second set asked 6 questions, which were made available periodically for a trading period of 2 weeks each. At the trading onset, each participant received 10,000 virtual credits for each question, with options to buy or sell the contracts. Each question had 6 prediction options and useful links to wiki articles and websites to inform trading choices (Figure 4). Each question had a judgment rule—how the result was determined—predetermined before trading was opened. In addition, participants received weekly texts and reminders to encourage participation.

#### System Design

Questions on COVID-19 outcomes were judged against numbers reported in the Ghana Health Service dashboard. Rewards were assessed at the end of the trading period based on the virtual credits left for each participant. The total virtual credits at the end of the trading period were tallied, and rewards were distributed per percentile placement, that is, those in the highest 10 percentile earned the highest rewards, and the rewards decreased as placement in the percentile was further from the top 10 percentile.

#### Rewards

**Figure 5:**
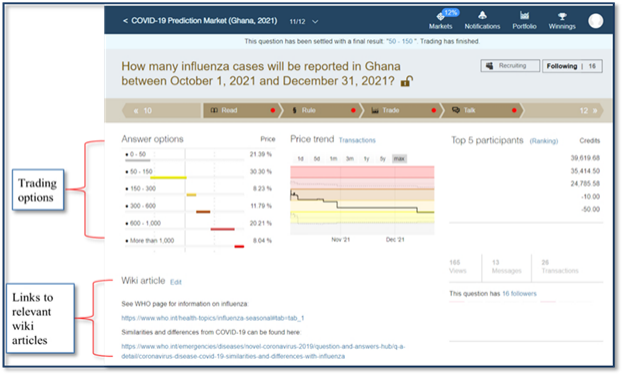
An example of how trading results are compared to the official reports for the period after the trading period to determine reward distributions.

Participants received rewards in phone credits ranging from GH ¢2.7 to GH ¢134.1 (US $0.23 to US $11.5) worth. Trade results are compared to the official reports for the period after the trading period to determine reward distributions (Figure 5). After buying and selling, participants could post the reasons for their trade choices in an interactive feature called “market talk.” This ensured higher participation and allowed participants to make a case for their trade (prices rose as more participants made the same trade).

### Data Collection

#### Overview

Participants completed a self-administered questionnaire before trading onset. The survey collected data on participant age, category of participants (health worker or community volunteer), employment status, and main source of information (radio, television, social media, other web-based platforms, and word of mouth), and COVID-19 knowledge. Data on trading frequencies were also extracted directly from the Prediki system.

A semistructured interview guide was used to explore the concepts of feasibility, user acceptability and engagement, changes in their health behaviors, and perceived COVID-19 risks related to the prediction markets used at the study’s exit. The interviews followed a chronological format, with questions centered around the participants’ experience before, during, and after participating in the activity. Participation in the in-depth interviews was open to anyone who had traded on the platform.

#### Process Evaluation

A total of 3 research team members were simultaneously interviewed to understand their perspectives on the implementation process of the IM activity. The interview guide was tailored to speak to the roles of these individuals. Rather than focusing on a chronological format—before, during, and after participation in the activity—the guide focused on stages in the implementation process: interest in the implementation process, planning, recruitment, execution, and reflection. The complete interview guide for this implementation team can be found in Multimedia Appendix 4. Key insights were added to an existing memo of the participant interviews.

### Statistical Analysis

Descriptive statistics were used to summarize the baseline characteristics of recruited participants. We also conducted a trend analysis to assess if there was a day-of-the-week effect per time of the day (average by day) on trading frequency. Ordinary least squares regression analysis was conducted to determine the factors associated with trading at least once. All statistical analyses were conducted using STATA (StataCorp LLC) software.

Qualitative data were thematically analyzed. A codebook developed for these interviews included 3 parent codes: 1 of a topical nature, 1 focused on the participant experience, and 1 focused on a participant’s journey. The journey parent code had 5 subcodes at the following levels within a participant’s journey: awareness, consideration, conversion, loyalty, and advocacy (Table S1 in Multimedia Appendix 4). The codes and transcripts were added to ATLAS.ti v3.16.1 (ATLAS.ti Scientific Software Development GmbH) for the coding process. Journey maps are a human-centered approach to capturing and sharing a person’s experience of learning about something (awareness), trying a solution (consideration or convert), and championing a solution (loyalty or advocacy) [31]. After the initial compilation of interview results, a high-level summary of each stage in the participant journey map as well as an ideal platform journey were shared with a few select participants in a focus group setting. This web-based process was to complete a “return of results,” explaining the results of the initial findings of the qualitative interviews back to the participants and adding any additional insights to these results to contribute to this iterative process and study design.

### Measures

The primary outcome was feasibility and participant acceptability. This was assessed using platform use metrics (market-specific hit rates) and qualitative interviews after the study’s conclusion. The secondary outcome was prediction market acceptability, which was qualitatively explored based on participants’ opinions and perceptions of the prediction market system from experience.

### Ethical Considerations

The Ghana Health Service Ethics Review Committee reviewed and the Institutional Review Board at The University of North Carolina at Chapel Hill approved this study (IRB 21- 3117). An informed consent form and intake survey were administered on the web through Qualtrics. Links were sent to all eligible participants. Participants were rewarded with their payoffs in the market via virtual credits on the Prediki system. These credits were converted to phone minutes and credited to participants’ accounts. Participants were rewarded as each contract was resolved (determined by how closely the prediction matched actual outcomes, as reported by the Ghana Health Service on the target date of the prediction).

## Results

### User Statistics

#### Overview

Of the 105 participants enrolled and sent links to trade on the IM platform (Prediki), 36 (34.3%) completed the baseline survey. The average age of the participants recruited was 28 (SD 5.2; range 20-45) years. Half of the participants (19/36, 53%) had received at least 1 COVID-19 vaccine shot, 75% (27/36) were sure they did not have COVID-19 at the time of the study, and all participants (36/36, 100%) had high COVID-19 knowledge (Table 1).

**Table 1:** Descriptive summary showing the baseline characteristics and COVID-19–related knowledge of participants who completed the baseline survey in Ghana in 2021 (n=36).

Factors		Values
Age (y), mean (SD)		29 (5)
Health workers^a^, n (%)		18 (50)
**Has a doctor or another health care professional diagnosed you with COVID-19?, n (%)**		
	No	33 (92)
	Yes	1 (3)
	Prefer not to answer	2 (6)
**Do you think you have been infected with COVID-19?, n (%)**		
	No	27 (75)
	Unsure	7 (19)
	Yes	2 (6)
**Have you received at least 1 dose of a COVID-19 vaccine?, n (%)**		
	Yes	19 (53)
	No	17 (47)
**COVID-19 can spread through mosquitoes, n (%)**		
	True	4 (11)
	False	32 (89)
**SARS-CoV-2 can live on certain surfaces for days, n (%)**		
	True	29 (81)
	False	5 (14)
	Unsure	2 (6)
**Antibiotics are effective at preventing COVID-19 infection, n (%)**		
	True	10 (27.8)
	False	18 (50.0)
	Unsure	8 (22.2)
**COVID-19 can spread through droplets coughed out by an infected person, n (%)**		
	True	36 (100)
	False	—^b^
**Blisters or sores on the throat are not symptoms of COVID-19, n (%)**		
	True	10 (28)
	False	18 (50)
	Unsure	8 (22)
**Washed hands with soap or used hand sanitizer several times a day, n (%)**		
	Yes	36 (100)
	No	0 (0)
**Avoided public spaces, gatherings, or crowds, n (%)**		
	Yes	29 (81)
	No	7 (19)
**Avoided contact with people who could be high risk, n (%)**		
	Yes	30 (83)
	No	6 (17)
**Canceled or postponed travel for work or pleasure, n (%)**		
	Yes	17 (47)
	No	19 (53)

^a^Health workers include nurses, doctors, laboratory technicians, physician assistants, midwives, and orderlies.

^b^Not applicable.

#### Traders Versus Nontraders

Most trading and nontrading participants were aged ≥30 years (13/19, 68% vs 53/86, 62%; *P*=.80) and health care workers (11/19, 58% vs 44/86, 51%; *P*=.59). Most participants who traded mainly obtained their COVID-19–related information from web-based platforms and social media (12/19, 63%), whereas most nontraders mainly obtained their information from multiple sources (41/86, 48%; Table 2).

**Table 2:** Differences in characteristics between the recruited participants who traded at least once on the Prediki system during the 3-month active trading period in 2021.

Factors		Traded at least once, n (%)		*P* value	
		No (n=86)	Yes (n=19)		
**Age (y)**					.58
	<30	33 (38)	6 (32)		
	≥30	53 (62)	13 (68)		
**Occupation**					.59
	Health worker^a^	44 (51)	11 (58)		
	Nonhealth worker^b^	42 (49)	8 (42)		
**Main sources of COVID-19–related information**					.09
	Internet^c^ or social media	33 (38)	12 (63)		
	Television or radio	12 (14)	3 (16)		
	Multiple sources^d^	41 (48)	4 (21)		

^a^Health workers include nurses, doctors, laboratory technicians, physician assistants, midwives, and orderlies.

^b^Nonhealth workers include students, unemployed persons, market traders, software engineers, bankers, drivers, and computer programmers.

^c^Internet includes news websites, search engines, blogs, and scientific publications.

^d^Multiple sources include web-based and offline sources, paper graphics, and word of mouth from others.

#### IM Uptake

Of the 105 participants invited to trade on the platform, 21 (20%) traded at least once. A total of 321 trades were made by the 21 participants across 12 questions in the platform. Questions estimating the number of COVID-19 cases at the regional level (for the Greater Accra region) received the least number of trade activities (range 6-13 trades). Comparatively, questions were used to estimate the national-level number of COVID-19 cases, and the final bonus question received the highest number of trades (range 13-19 trades). Multimedia Appendix 5 presents a detailed summary of trading activities for each question and the number of rewards paid out per question.

#### Factors Associated With Trading

Participants who obtained their COVID-19–related information mainly from television or radio were less likely to participate (marginal effect: −0.184) than those who obtained information from social media or internet. However, if they did trade, those who got information from television or radio made more trades and earned more rewards. Among those who traded, those aged <30 years made 7.5 times more trades than those aged >30 years (marginal effect: 0.0135) and earned GH ¢134.1 (approximately US $11.7) more in rewards. There was no substantial relationship between being a health worker and participating in any trade or among those who traded regarding the number of questions traded and rewards earned. Table 3 presents the details of the ordinary least squares regression estimations with robust SEs.

**Table 3:** Ordinary least squares (linear probability model) regression results showing factors associated with COVID-19 prediction markets trading uptake and frequency among Ghanaian participants in 2021 (n=21).

Variables		Any trade made, OLS^a^	Number of questions, OLS	Rewards, OLS
**Age (y)**				
	<30	0.0135	7.548	134.1
	≥30	1	1	1
**Occupation**				
	Health worker^b^	−0.0602	0.918	11.95
	Nonhealth worker^c^	1	1	1
**The main source of COVID-19 information**				
	Multiple sources^d^	−0.0912	3.688	81.30^e^
	Television or radio	−0.184^e^	4.671^f^	85.54^e^
	Internet^g^ or social media	1	1	1

^a^OLS: ordinary least squares.

^b^Health workers include nurses, doctors, laboratory technicians, physician assistants, midwives, and orderlies.

^c^Nonhealth workers include students, unemployed persons, market traders, software engineers, bankers, drivers, and computer programmers.

^d^Multiple sources include web-based and offline sources, paper graphics, and word of mouth from others.

^e^*P*<.05.

^f^*P*<.10.

^g^Internet includes news websites, search engines, blogs, and scientific publications.

### Qualitative Results

Overall, 10 (48%) of the 21 trading participants were engaged in our in-depth interview over Zoom (Zoom Video Communications Inc). Many factors influencing the feasibility of implementing a viable IM-based disease surveillance program were identified in this study. Key facilitators identified included having (1) a strong onboarding process through the WhatsApp platform, (2) a genuine curiosity and motivation to experiment with the platform, (3) interactive features within the platform, (4) peer influence, and (5) rewards. The main barriers identified included (1) difficulty understanding the trading process and (2) various IT issues and considerations. We found little evidence that participants changed their long-term COVID-19 risk mitigating behaviors.

### Facilitators

#### Strong Onboarding Process Through WhatsApp

A project-specific WhatsApp group was an important tool that immediately created a positive onboarding experience for participants and facilitated communication between participants and the research team. Participants could communicate directly with the designated support project staff if they had any specific questions. WhatsApp messages served as reminders for participants to check the digital platform. In addition, the introductory videos showing how to use the platform, trade, and earn more rewards were shared on the WhatsApp group, allowing participants to understand how to operate and navigate the platform, given that the videos were short and visual. Participants could also ask for technical support from designated staff on WhatsApp during the trading process and receive notifications about new open questions and trading reminders on the group page. This helped keep participants updated on questions open for trades and actively engaged in the IM process:

...The toggle video is the one with the cartoon. It was very helpful. Because I had my hands on many things, taking time to read was difficult, so I think the videos were helpful. You watch it in about 2 minutes and get the whole idea.

Participant 6

#### Curiosity About IMs

All participants had genuine curiosity, interest, and motivation to participate in this pilot. Of 10 participants, 6 (60%) heard about the activity from friends and chose to participate because their friends participated. Half of the participants (5/10, 50%) interviewed were curious about how IMs worked and wanted to learn more about the platform. In total, 20% (2/10) of participants wanted to understand the frequency of how COVID-19 was evolving in Ghana, and others wanted to share their ideas to give back to their community and help their country. Participating health care workers also expressed that this was a good way to share their voices on this topic as health professionals.

Furthermore, seeing other participants actively engaged on the platform motivated participants to continue trading. A total of 20% (2/10) of interviewees and some focus group discussion participants indicated that their motivation did not mainly stem from the novelty of platform features or the direct reminder messages from the administrator but rather from seeing the activeness of their peers on the platform. Moreover, understanding one’s genuine interest in this material was a precursor for their acceptability of the platform when assessing their willingness to participate in future IM surveillance in their daily life. Moreover, participants cited that persons who are genuinely curious about IMs and want to explore data (perhaps a student or someone interested in research) are the ideal participants to consider when deciding whether to share this platform with a friend:

I just wanted to do something different from what I used to do, to learn more and to share ideas, and as a health professional, I was thinking that maybe this was right for me to put out my voice concerning this particular new disease that has come either to stay or will go away in a short while.

Participant 3

#### Interactive Features Within the Platform

Besides the trading process, the platform (Prediki) had an interactive chat feature that participants enjoyed. Many participants did not see the chat capabilities and initially felt there needed to be more interaction on the platform. However, once they discovered this feature, it allowed for an interactive experience that encouraged sharing knowledge and diverse opinions. Many participants valued the diversity of thoughts shared in the community section on the platform and deemed it a key attractor to this type of trading activity. The competitive nature of the activity attracted some participants but deterred others. While some enjoyed the back-and-forth in the comments section and partly based their predictions on others’ opinions, others wished the platform was more collaborative and had participants working together more explicitly. The platform also had a ranking system that ranked participants based on how many points they had accrued from their predictions. Many participants appreciated this feature and saw it as a motivator to try and improve their predictions to increase their rank:

I found it interesting because, at some point, you will have conflicting ideas, you’re your colleagues, and at some point, you will realize that your other colleague will come in to support your explanation that you have given to the reason why probably you chose a particular range...it was a nice way of sharing knowledge.

Participant 1

#### Peer Promotion and Rewards

Some participants (4/10, 40%) heard about the activity through a friend or had a friend participate. This was a key facilitator in getting people to sign on to the pilot and for sharing information in the future. To scale up the platform use, all participants said they would recommend it to friends and family as those people would be more trusting and willing to try and iterate how they would describe the platform to others. Other participants proposed promoting the platform’s social and fun angle, while some advocated promoting the educational appeal. All participants agreed that social media should be explored to recruit more diverse participants. However, friends and family would be the starting recruitment point for future platform iterations.

In addition, rewards were deemed a strong motivator for participation in prediction markets. Several participants appreciated the phone credits but mentioned that a monetary reward would be more valuable than the phone credits. This is because some participants received phone credits through work; other participants already had beneficial phone credit bundle subscriptions. Therefore, cash rewards allowed for more flexibility in spending on other things they may need besides phone credit. Moreover, nearly all participants agreed that more active participants should receive more compensation for their time and efforts and there should be rewards for the referral of new participants:

...basically, I was contacted by a friend, my mate in school, who told me about it, and I did not have to think twice. I told him I was in, and that is where it started.

Participant 8

### Barriers

#### Trading and Technical Difficulties

Although all participants cited a willingness to participate again if offered, understanding the trading process could have helped active engagement and enjoyment of the experience. Some participants needed clarification on the trading process even after repeatedly watching the tutorial videos. This difficulty remained across every step of the trading process, from understanding the terminology of “confirming a trade” to the concept of “altering an existing trade.” Although the videos were helpful, 3 (30%) out of the 10 participants interviewed suggested having a “classroom training*”* session using Zoom or another platform to answer participant questions live as they watched the explanatory videos. Not only would this help answer any questions that participants may have, but as 1 (10%) participant pointed out, it would also help increase engagement in the overall activity. In addition, receiving a successful trade confirmation notification was recommended by both participants and research team members to resolve some of the confusion surrounding trading. Of 10 participants, 5 (50%) also suggested that providing detailed education and training on trading and how trading markets work earlier in the onboarding process would help people overcome these difficulties (Multimedia Appendix 4).

It was recommended that allowing for a dynamic onboarding approach where participants may communicate via oral language versus written in WhatsApp rather than static videos would provide a more educational experience. In addition, highlighting some difficulty-related questions in a frequently asked questions section on the platform for future reference was recommended. Some participants also mentioned the need for more visuals (“Maybe some videos, 2 minutes, 1-minute video” [Participant 8] and “colored and boldened so that when you get to the platform, you know that this is what you are talking about...” [Participant 7]), short excerpts, or short voice notes with detailed explanations on trading to offer a broad set of learning options. The discrepancy between points and the actual monetary value of the points earned also confused some participants as they “had no idea what they would earn” and ended up discouraged when their points gained largely exceeded the financial reward received. Implementation team members also mentioned that disseminating the rewards was cumbersome and could be streamlined in the future.

#### Unidirectional Communication

Besides the technical specifications of the onboarding process, participants noted that it would have been nice if members were lively and more interactive with each other from the start. Initially, the WhatsApp platform was only set up for 1-way communication (ie, from the administrator to the participants as a group). Although participants could directly message the administrator for help, many participants would have liked 2-way communication with other participants on WhatsApp. Many participants also emphasized the need for a more mobile-friendly platform in the future, as many participants engaged with the website through their laptops. Only 1 (10%) participant was technology savvy enough to interact with the site on their mobile device by keeping a window tab open in the web browser. However, not everyone would understand how to do this. Therefore, creating an app would allow for a smoother participant and referral experience (Multimedia Appendix 4).

#### Low Participation

Although 105 participants were recruited, only 21 (20%) traded on the platform. Of the 10 participants interviewed, 2 (20%) mentioned that their busy schedules hindered greater participation. Others may need constant reminders to remember to trade, as many Ghanaians do not check their email regularly. Therefore, 3 (30%) of the 10 participants suggested a “notifications” feature to keep people engaged daily, besides the WhatsApp reminders for a future iteration of the platform. More specifically, 1 (10%) of the 10 participants highlighted the need for notifications sent via a message (such as SMS text message or push notifications), call, or app notification, instead of directing people to their email, recognizing the effects of limited data use, particularly in settings with poor telecommunication services. The implementation team members also noted that addressing password recovery as a high-return but low-effort task would remarkably improve a participant’s experience with the platform:

It was cool. I was very active from the beginning, but right after the end, I was not that active because I was doing my internship and helped my mom take care of me in school. So, I was working two jobs at the same time.

Participant 4

#### Competition With Other Web-Based Spaces and Pilot Delays

Although we thought participation rates would have been higher considering COVID-19’s impact and global acceleration into the virtual world, this acceleration also increased the competition for people’s time in other spaces (such as social media and dating apps). Another implementation team member also mentioned the long delay between the initial participant recruitment and onboarding to the platform. Therefore, the curiosity about IMs and interest in how they work would have withered when the platform was finally opened for trading. This may have contributed to lower participation rates. One of the research team members narrated that “several individuals had forgotten why the administrator was reaching out to them as a length of time had passed between being recruited and the actual launch of the platform.” Although this delay is not unusual when developing and piloting new systems, the duration between recruitment and opening of the platform for trading can be reduced by ensuring that recruitment and training only commence when the platform is actively ready for use. In addition, establishing a social media group to keep participants engaged through demonstrations and bidirectional communication would have kept participants actively engaged during such waiting periods.

## Discussion

### Overview

Traditional infectious disease surveillance is challenging and prohibitively costly in many LMICs, increasing the importance of developing new infectious disease surveillance methods. Participatory disease surveillance offers a cheap and flexible option but is vulnerable to multiple issues that limit their ability to inform disease response. Designing participatory disease surveillance systems using IMs can address many of these challenges, and IM-based participatory disease surveillance has been successfully piloted in high-income contexts [19,24]. Our data suggest that prediction markets were feasible for engaging local Ghanaian communities in COVID-19 control responses. We also found that user feedback could be used to improve the prediction market platform. This study extends the literature by focusing on an LMIC setting and iteratively developing the prediction market with end users using participatory methods.

### Principal Findings

Our results demonstrate that using IMs for infectious disease surveillance is feasible in Ghana. This finding is consistent with a small amount of literature on using IMs to enhance infectious disease surveillance [19,23-25]. Most prediction markets for infectious diseases have focused on the United States and other high-income countries, neglecting resource-limited settings [23]. This may result from more familiarity with information or prediction markets in high-income settings or fewer digital health resources in LMICs. Designing an IM system as an ad hoc structure to complement disease surveillance or integrating it into existing health monitoring structures could expand public health surveillance in resource-limited countries. IMs could effectively and efficiently capture real-time data to accelerate policy and planning. Further research is needed to scale up this approach and assess its effectiveness in tracking disease trends.

The qualitative findings from our study showed how social innovation approaches could be used to improve prototypes based on local feedback iteratively. Social innovation is a community-engaged process that links social change and health improvement [32]. Our findings corroborate this, which suggested that prediction market systems could facilitate community participation in public health monitoring, but the system design is crucial to increasing community engagement. Thus, the ability of the target population to use digital tools at varying levels, the cost of internet services, and internet coverage must be critically considered when determining the design and features of a prediction market system for LMICs. In addition, researchers should consider designing IM systems as applications that can be supported by multiple mobile phone operating software (eg, Androids, iOS, and Windows) and web browsers to cater to users of varying levels of technology savviness.

Our data also confirm observations of other studies demonstrating that IM surveys could effectively enable community participation in real-time infectious disease monitoring. IMs provide a formal mechanism to aggregate views of diverse individuals to generate forecasts that could improve response to infectious disease emergencies [19,33]. This approach may allow individuals with low income, ethnic and racial minority groups, and others to contribute to infectious disease control efforts. However, our data also highlights the need for further platform refinement. Many participants found some aspects of the user interface duplicative and not intuitive, making navigation difficult. In addition, other methods to decrease the digital divide and ensure broad access are needed. For example, an unstructured supplementary service data interface could allow people to participate without a smartphone.

Low participation could hinder the successful use of IMs for infectious disease surveillance in LMICs. The participation rate that we observed (21/105, 20%) is similar to the findings of another study, which reported that only 20% of their study participants accounted for 80% of trades made during the season under review [24]. Despite this, relatively low take-up may be less consequential for IM-based systems than for more traditional participatory disease surveillance systems [18]. Therefore, IM surveillance data could complement traditional participatory disease surveillance and reduce the impact of the lack of population representativeness. Moreover, there are multiple ways in which participation can be increased, such as tweaking platform design, enabling more engagement, and offering optional notifications and trading updates.

Beyond implementation in an LMIC, our study built on the existing literature by expanding the sample population to a broader set of participants (including general community members in addition to health care professionals) and included human-centered design thinking principles in the methods to better capture participant experience. Although this study was not designed to evaluate the quantitative results of the IM as they related to existing surveillance methods, which is left for future studies, our pilot data have implications for research and policy. From a research perspective, this study shows that IMs for infectious disease surveillance are feasible in LMIC settings. On the basis of the feedback from the participant and implementation team interviews and focus group discussions, we have compiled the ideal participant journey map for a future version of an IM-based platform (Figure S1 in Multimedia Appendix 4). Further research is needed to ensure broader engagement, especially among people with limited technology experience. In addition, randomized controlled trials are needed to assess the effectiveness of prediction markets compared to conventional case-based surveillance systems. From a policy perspective, there is a need for technological and regulatory support to create IMs, and the availability of public data on reported infectious diseases is necessary.

### Limitations

Our study has limitations. First, our sample size is small, and therefore, the views and experiences of the study participants are not representative of the general population. However, our pilot study provides preliminary data and lessons to inform subsequent research into using predictive markets for infectious disease surveillance in LMICs. Second, specific characteristics of the prediction market system may have limited engagement and limited our ability to use the data to estimate COVID-19 cases. Although the pilot was implemented with a computer default, several users accessed the platform on their smartphones. Subsequent studies should consider adopting smartphone-based designs for user convenience to mitigate this limitation and encourage community engagement. Subsequent studies should also consider having social media–based chat groups where participants can directly interact with each other to make their experience more enjoyable. Third, IMs are limited in the context of capturing the syndromic profiles of reporting individuals. Therefore, IMs should be used to complement data from traditional surveillance and passive disease surveillance systems but not as replacements for those systems.

### Conclusions

This study contributes to the growing literature around participatory disease surveillance approaches by demonstrating the feasibility of tailoring existing systems for use in LMIC settings, where they are urgently needed. Our findings showed that using IM surveillance to supplement traditional laboratory and syndromic monitoring systems is feasible and acceptable in LMICs. Our qualitative findings also better explained the barriers and facilitators of implementing an IM-based program. Moreover, the ideal journey framework we have developed could inform future implementation designs. However, adequate education on trading and a more user-friendly technological interface with offline notification systems are needed to ensure optimum participation in future IMs. In addition, ensuring a comprehensive onboarding process with bidirectional communication and social media promotion platforms is essential to keep participants continuously engaged and interested in prediction markets. Future research should incorporate greater equity in the sample population and explore variations in geographic diversity to fully understand the potential of IMs in daily national disease surveillance systems of other LMICs.

## Appendix

Multimedia Appendix 1 Video introduction on COVID-19 prediction markets surveillance to complement efforts of the Ghana Health Service in monitoring COVID-19 outbreaks during the pandemic.

Multimedia Appendix 2 Video showing the step-by-step process of trading participation in the COVID-19 prediction markets survey on the Prediki platform.

Multimedia Appendix 3 Video showing the trading rewarding process and how rewards are determined in the prediction markets survey.

Multimedia Appendix 4 Supplementary tables, explanatory texts, participants excerpt quotes, thematic coding tree, and a figure showing the ideal user journey.

Multimedia Appendix 5 The questions used in the Prediki prediction markets surveillance for Ghana on the Prediki platform and the frequency of trades made by participants per question for 3 months in 2021.

## Abbreviations

IM: information market
LMIC: low- and middle-income country
