# Supplementary material for: Collective Intelligence–Based Participatory COVID-19 Surveillance in Accra, Ghana: Pilot Mixed Methods Study
Source: JMIR Infodemiology. 2024 Aug 12;4:e50125. doi: 10.2196/50125 (PMC11347900; doi:10.2196/50125)
Supplement: Multimedia Appendix 4 [file infodemiology_v4i1e50125_app4.pdf]

SUPPLEMENTARY FILES LEGEND

Material 1: Excerpt quotes from in-depth interviews and focus group discussions.

Material 2: Video introduction on Covid-19 prediction markets surveillance to complement efforts of the Ghana health service in monitoring Covid-19 outbreaks during the pandemic.

Material 3: Video showing the step-by-step process of trading participation in the Covid-19 prediction markets survey on the Prediki platform.

Material 4: Video showing the trading rewarding process and how rewards are determined in the prediction markets survey.

Material 5: Informed consent and complete interview guide.

Table S**1:** Detailed Codebook

Figure S**1:** Shows an ideal 'participant journey map' informed by participant feedback from their experience in using the prediction markets platform.

Figure S**2:** Shows association between prediction markets questions, days of the week posted and trading frequency from October to December, 2021.

Material 1

Excerpt quote 1:

“I don't know whether I am having the words right; we have long against and shortfalls. Personally, I don't know but I didn't really understand what these two variables were doing" (P1).
“I wasn’t sure whether when I pick whether I really traded or not… I think there should be something that will be telling me that you have successfully traded” (P3).
“As we are all watching, if you have a question, then you ask, so I think that session would have made people more interested, it would have ignited their interest to see how the whole process would go, and it would have made their trade more, that is what I feel.” (P10)

Excerpt quote 2:

“The challenge is that the WhatsApp group has been muted for everybody except the platform host, so basically, if you have a question, you cannot ask, and for me, I think it kind of takes some interest for me.” (P8)
“I will say that if we can get a link that will direct the user to an app so that when the person just connects, the person gets directed to a mini app on his phone, a short app, a short desktop icon where the person can access it now and then, I think it will be of a great help than the person just going to open a browser or an email now and then.” (P6)
“Not getting a notification about any updates or if someone commented unless I personally log in back to check. So, I had to use email to alert me anytime someone commented on the page.” (P4). “In Ghana most of us, even those who claim we are educated, we do not mostly check our emails. We like things to be sent to us personally, maybe through text message or a call.” (P10).
“Sometimes I have other apps, I off my data, but I still get push notifications on the apps telling me something, and then I switch on the data, I go back to the apps, and then when I am done, I switch it off again, that is what I am thinking.” (Focus group)

Material 5

Interview Guide

Informed Consent

Brief Introduction

• Introduce ourselves

• Why are we talking to them?

o "We would like to take a few minutes of your time to talk about the recent online prediction market you participated in. We want to understand what worked and what did not work for you so we can improve it in the future."

• Have the participant introduce themselves (e.g., name, how they spend their days, what they do outside of work etc.)

• Confidentiality

o "We will be sharing these findings with the research team that designed the prediction market activity, but your name and any identifiable characteristics will not be shared with anyone in the process. Additionally, you do not have to answer any questions you do not feel comfortable answering."

• "Before we get started, do you have any questions for us?"

"Before Participating" (Reference to time anchor for participant)

• What were you doing to reduce your risk of getting COVID-19 before you participated in this activity?

1. Probe: handwashing, social distancing, wearing a mask etc.

• Have you or any family members been affected by COVID-19? If yes, how?

• Why did you choose to participate in this prediction market activity?

1. Probe: What is motivating you to continue?

"While Participating" (Reference to time anchor for participant)

• What does Prediki do?

• Please tell me about your experience with the initial onboarding videos and instructions that were shared with you via WhatsApp. How helpful or useful were these?

• What was your experience like using the Prediki platform?

1. Probe: What did you think about the chat and ranking features in the platform?

2. Probe: What did you like about the platform? What did you not like?

3. Probe: What challenges did you face while trading on the platform?

• If you had a magic wand, what would you change about this platform to make it better? Why?

• What data sources did you use to help with your predictions?

• While you were participating in the pilot, how did your normal routine change because of COVID-19?

1. Probes: Did you start doing or stop doing certain activities because of COVID-19? Like traveling, going to work or school, meeting up with friends and family.

<u>“After Participating” (Reference to time anchor for participant)</u>

• Would you use Prediki again? Why or why not?

1. Probe: What would keep you interested in using this more or again?

• Would you recommend Prediki to a friend? Why or why not?

• What would be the best way to share info about Prediki with others?

1. Probe: Who would you trust to advertise this platform to you?

• So, you know this exercise was to see if the prediction market platform is feasible in predicting COVID-19 related outcomes and helping combat future outbreaks. What did you think of the trading process in general?

1. Probe: How would you make it easier for people to trade on the platform?

• How much did the “rewards” factor into your decision to participate in the program?

1. Probe: In your opinion, what would be the best form of reward for this activity? Why?

• What suggestions do you have for motivating others like yourself to use this platform or a platform like it?

• After participating in this pilot, would you say your knowledge on COVID-19 has changed?

1. Probe: If so, how? or if not, why not?

<u>Dissemination</u>

1. Would you be interested in participating in a second interview in a few weeks? We plan to compile information from these first-round interviews into a summary report and would like to co-create an improved solution with you.

2. After this is finished, would you be interested in seeing a final version of the report?

Table S1: Detailed Codebook summary showing definitions and examples of the five subcodes following levels within a participant's journey.

Parent Code	Parent Code #	Subcode	Subcode #	Apply this code when participants…	Example Quote
Topical	1	COVID risk mitigating behaviors	1.1	Discuss what COVID risk mitigating behaviors they were doing, either before, during or after participation in the Prediki activity	"The nature of my work was not allowing me to stay home so I had to go outside but one thing I try to do is to try and wear the mask and try to avoid crowded areas"
Topical	1	Motivation	1.2	Describe why they chose to participate in this activity or why they continued to participate	..."this was something that was giving me the opportunity seeing the way covid is involving every 2 weeks especially with the vaccination and other measures that were put in place. That actually motivated me"
Topical	1	Data sources	1.3	Explain what data sources they used to make their predictions	"Yes, I normally use the Ghana Health Service"
Topical	1	Rewards	1.4	Mention anything regarding the monetary rewards of participating in the Prediki activity	"I think to be fair those who are participating more need to be rewarded because I felt some people normally come in and feel that let me just select something and leave without really understanding the trend of covid "
Experience	2	Feasibility	2.1	Discuss elements that helps or hinders a user's ability to use the Prediki platform (e.g., Wi-Fi access, onboarding process -	"In Ghana most of us, even those who claim we are educated, we don't mostly check our emails. We like things to be sent to use personally, maybe through text message or a call"

Experience	2	Acceptability	2.2	Discuss their willingness or unwillingness to use the Prediki platform in their everyday life	"There are certain people that I don’t mind, those who are into more of research and data analysis. They are those who will enjoy doing stuffs like this"
Experience	2	Usability	2.3	Discuss their experience using the Prediki platform (e.g., features, what they liked/did not like, usability of it) and using it for its intended purpose	"For instance if I am part of the first 3 or the first 5 or the first 10, I can see where I am but if I am part of the, if I am on 52nd position, I am not able to see my potion so I think this should be done in ranges of 5, ranges of 10 so that anywhere the person falls, the person will be in that range"
Experience	2	Pain points	2.4	Describe negative experiences / interactions within the Prediki activity	"Initially what I didn’t like, or I didn’t understand, later on I realized that I didn’t understand it. It was after making a prediction you realize that you will be having a negative figure attached to your investment"
Experience	2	Delights	2.5	Describe positive experiences / interactions within the Prediki activity	"It makes me to always try to call my family, that is when I am not there and I am in Kumasi, call them and try to educate them to be extra careful because these are the numbers that I am seeing. The reason why it was helpful is because Ghana Health Service was not reporting the figures again on the national television. They were rather now doing it on their data base meaning if you don’t go to their database, you might not be able to know what is actually happening "
Journey	3	Awareness	3.1	Discuss if they had heard of, used Prediki prior to this activity or how they would be	"Okay so truthfully I only heard the name Prediki, and it was from one econ platform that I joined some time ago. It was not more than a year but in the last 8

				open to hearing about it in the future	months or so, but I didn’t really know what they actually do so when I saw the link again, I was like let me try and scroll through it "
Journey	3	Consideration	3.2	Describe the onboarding process and their initial experience with the platform	"Okay so I will say it was useful but many of us at the initial stage didn’t really understand what was actually going on, so it was later that I saw Emmanuel putting on the platform some videos"
Journey	3	Convert	3.3	Describe the extent to which they participated in the Prediki activity	"Because sometimes I normally try to answer or predict mine during the first 3 days or 4 days of the new question but sometimes, I forget that the 2 weeks is up so anytime he puts the reminder there then I will be like oh there is a new question and I have to go and look at the question"
Journey	3	Loyalty	3.4	Describe if they would use Prediki again or what it would take for them to use it again	"If I have the chance, I will definitely use it "
Journey	3	Advocacy	3.5	Describe if they would recommend Prediki to a friend and if so, how they would share information about Prediki	"One way I can do that is probably share the link to my friends. Actually, I started doing that because some of my friends were asking me, you are always reading about covid and then make some predictions, what are you doing so I told them about Prediki and the prediction market."

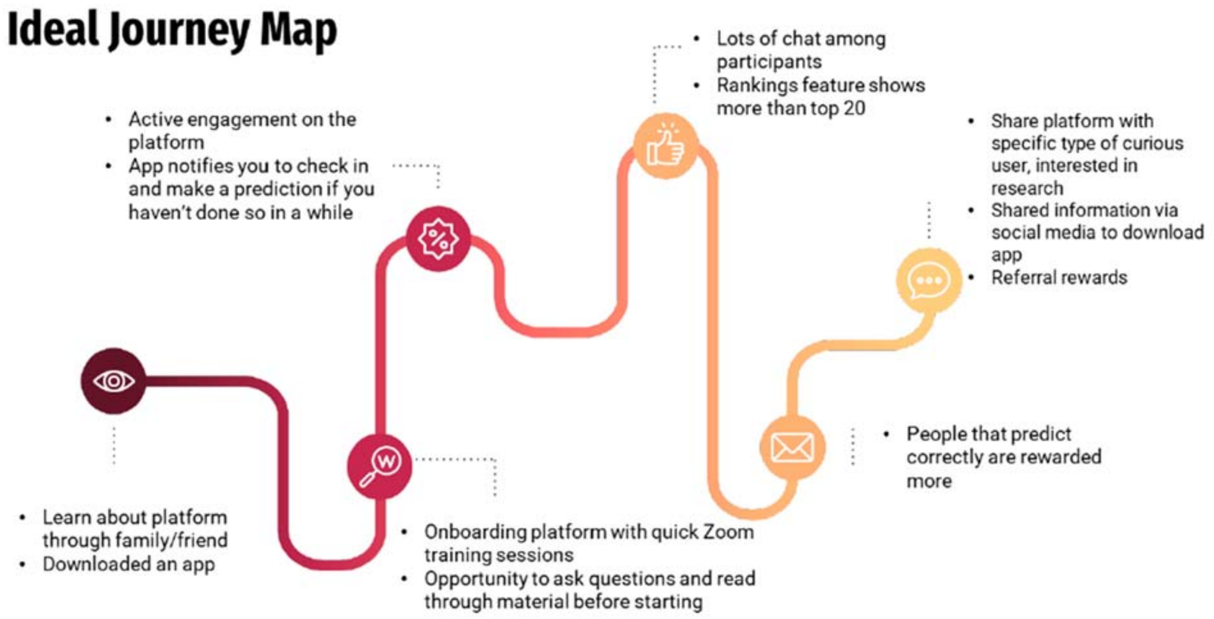

Figure S1. Shows an Ideal Participant Journey Map informed by participant feedback from their experience in using the prediction markets platform.

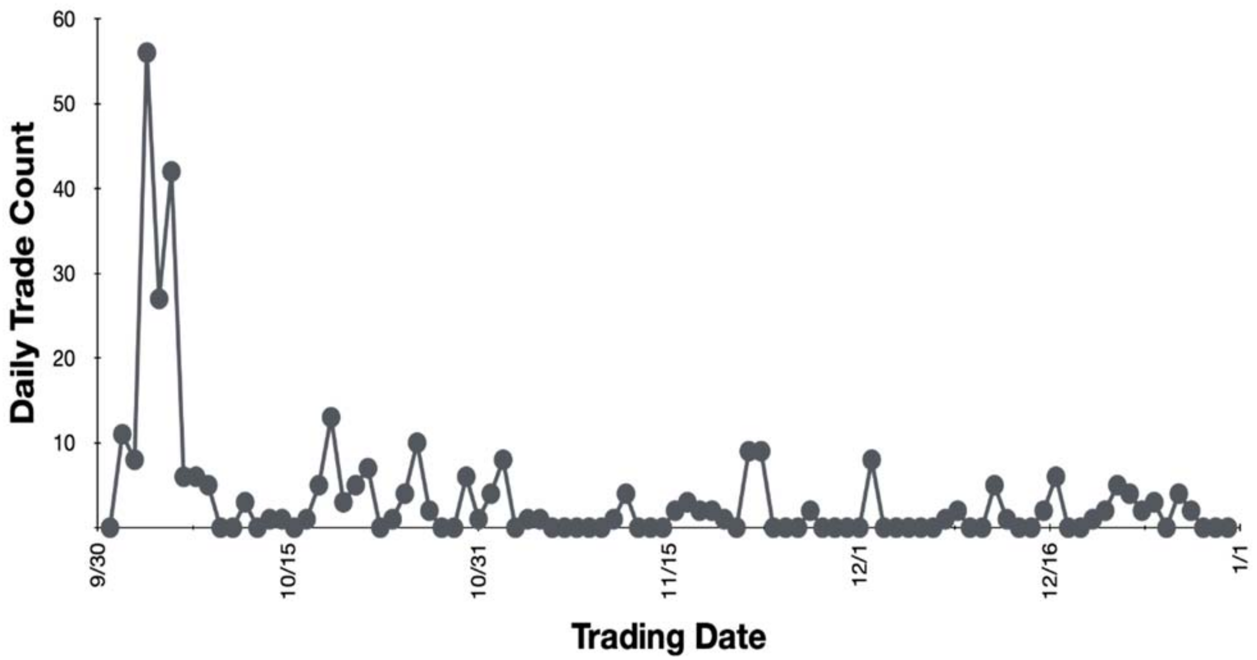

Figure S2. Shows association between prediction markets questions, days of the week posted and trading frequency from October to December, 2021.
