# Supplementary material for: Collective Intelligence–Based Participatory COVID-19 Surveillance in Accra, Ghana: Pilot Mixed Methods Study
Source: JMIR Infodemiology. 2024 Aug 12;4:e50125. doi: 10.2196/50125 (PMC11347900; doi:10.2196/50125)
Supplement: Multimedia Appendix 5 [file infodemiology_v4i1e50125_app5.docx]

**Multimedia Appendix 5.** The questions used in the Prediki prediction markets surveillance for Ghana on the Prediki platform and the frequency of trades made by participants per question for 3 months in 2021.

| Question | | Reference data source | Transaction period | Trades (n=21), n | Transactions (n=321), n | Market comments (n=140), n | Rewards distributed (n=2199), n |
| --- | --- | --- | --- | --- | --- | --- | --- |
| **COVID-19 year-end 2021 statistics** | | | | | | | |
|  | How many total (cumulative) COVID-19 cases will be reported for the Greater Accra Region by December 31, 2021? | GHS^a^ | October 1 to December 31, 2021 | 15 | 31 | 16 | 253 |
|  | How many total (cumulative) COVID-19 cases will be reported in Ghana (nationwide) by December 31, 2021? | GHS | October 1 to December 31, 2021 | 19 | 67 | 24 | 313 |
|  | How many individuals in Ghana will have received at least 1 dose of a COVID-19 vaccine by December 31, 2021? | GHS | October 1 to December 31, 2021 | 13 | 28 | 13 | 216 |
|  | How many total (cumulative) COVID-19 deaths will be reported in Ghana (nationwide) by December 31, 2021? | GHS | October 1 to December 31, 2021 | 14 | 30 | 16 | 233 |
|  | How many influenza cases will be reported in Ghana (nationwide) between October 1 and December 31, 2021? | FluNet system | October 1 to December 31, 2021 | 13 | 26 | 13 | 210 |
| **Greater Accra biweekly COVID-19 cases (condense)** | | | | | | | |
|  | How many new COVID-19 cases will be reported for the Greater Accra Region between October 1 and October 15, 2021? | GHS | October 1 to October 15, 2021 | 13 | 25 | 17 | 187.5 |
|  | How many new COVID-19 cases will be reported for the Greater Accra Region between October 16 and October 31, 2021? | GHS | October 16 to October 31, 2021 | 9 | 15 | 12 | 79.5 |
|  | How many new COVID-19 cases will be reported for the Greater Accra Region between November 1 and November 15, 2021? | GHS | November 1 to November 15, 2021 | 9 | 14 | 7 | 81 |
|  | How many new COVID-19 cases will be reported for the Greater Accra Region between November 16 and November 30, 2021? | GHS | November 16 to November 30, 2021 | 7 | 3 | 3 | 70.5 |
|  | How many new COVID-19 cases will be reported for the Greater Accra Region between December 1 and December 15, 2021? | GHS | December 1 to December 15, 2021 | 9 | 14 | 5 | 79.5 |
|  | How many new COVID-19 cases will be reported for the Greater Accra Region between December 15 and December 31, 2021? | GHS | December 16 to December 31, 2021 | 6 | 18 | 1 | 56 |
| **Bonus question (unsettled)** | | | | | | | |
|  | What percentage in Greater Accra will respond “yes” to the question, “In the last 30 days, have you skipped a meal due to lack of money or resources”? | Social media survey | October 1 to December 31, 2021 | 14 | 23 | 13 | 420 |

^a^GHS: Ghana Health Service.
